# Poisoning by Cocaine

**Published:** 1892-04

**Authors:** 


### POISONING BY COCAINE.

Berger (*Gazette des Hopitaux*, 1881, p. 1367) reported a case of poisoning by cocaine at a recent meeting of the French Society of Surgery. The patient was a healthy young man, with a hydrocele the size of a turkey's egg, very tense, which was tapped and evacuated, and a spoonful of a two-per-cent. solution of cocaine (estimated to contain forty centigrammes—about seven grains—of the alkaloid) injected and at once allowed to run out, apparently the whole quantity being recovered. An injection of tincture of iodine was then made into the sac, and the patient walked out, but soon returned complaining of feeling ill, and falling into collapse, in spite of vigorous treatment, he shortly died in coma preceded by convulsions. The autopsy revealed no other cause of death, and nothing to account for the patient's idiosyncrasy. The hydrocele-sac did not communicate with the peritoneal cavity.

In the discussion which followed, two other cases of accidents after such injections for hydrocele were reported, but they were not fatal. Reclus claimed that accidents would not happen if not more than ten centigrammes (about one and one-half grains) of cocaine were employed, and he had himself operated over sixteen hundred times without any accidents. Pozzi had seen an alarming condition follow an injection of two and one-half centigrammes (less than half a grain) into the gum to open an alveolar abscess. The reviewer feels compelled to add that with such large doses as are employed in the fatal case, and other cases mentioned in the discussion, the alarming symptoms are not surprising.—(*International Med. Mag.*) March, 1892, p. 194.

